# The Effectiveness of Prophylactic Compression Sleeves in Reducing the Risk of Lymphedema in Patients Who Receive Breast Cancer Surgery: A Systematic Review

**DOI:** 10.3390/curroncol32120660

**Published:** 2025-11-25

**Authors:** Sarah Shahid, Amanda Moerahoe, Gabriel Boldt, Allison Maciver

**Affiliations:** 1 Department of Surgery, St. Josephs Hospital, London, ON N6A 4V2, Canada; 2 University Health Network, Toronto General Hospital, Toronto, ON M5G 2C4, Canada; amanda.moerahoe@uhn.ca; 3 Departments of Surgery Oncology, London Health Science Centre, London, ON N6A 5W9, Canada; gabriel.boldt@lhsc.on.ca; 4 Departments of Surgery and Oncology, Western University, London, ON N6A 3K7, Canada; allison.maciver@lhsc.on.ca

**Keywords:** breast cancer surgery, lymphedema prevention, compression sleeves, prophylactic therapy, systematic review

## Abstract

Lymphedema is a common complication after breast cancer surgery, often causing functional limitations and reduced quality of life. Prophylactic compression sleeves have been suggested as a preventive strategy, but their effectiveness remains unclear. We conducted a systematic review of randomized controlled trials to evaluate whether early use of compression sleeves lowers the risk of lymphedema in this population. Comprehensive searches of major medical databases were performed, and eligible studies were analyzed. The findings showed that prophylactic compression sleeves did not significantly reduce the incidence of lymphedema compared with standard care. Some studies reported smaller increases in arm volume among sleeve users, but these results were inconsistent. Overall, compression sleeves appear to have limited value for lymphedema prevention, though they may provide modest benefit in reducing early swelling. Further high-quality research is needed to establish clear recommendations for clinical practice.

## Simple Summary

Breast cancer surgery can sometimes damage the lymphatic system, leading to swelling in the arm known as lymphedema. This condition can cause discomfort, reduced mobility, and a lasting impact on quality of life. Compression sleeves are already used as a treatment once lymphedema develops, and some doctors have suggested using them right after surgery to try to prevent the condition before it starts. In this study, we reviewed all available trials that tested whether wearing compression sleeves after breast cancer surgery lowers the risk of lymphedema. We found that using the sleeves after surgery did not significantly reduce the number of people who developed lymphedema, although there was some evidence of less arm swelling in those who wore them. These findings highlight the need for larger and higher-quality studies to guide future recommendations and help patients and doctors make informed choices about prevention.

## 1. Introduction

Breast cancer-related lymphedema (BCRL) is a chronic condition that can develop after breast cancer surgery due to damage in the lymphatic system of the axilla [1,2]. BCRL is characterized by swelling and disfigurement in the ipsilateral arm, limiting function and mobility of the limb [3]. BCRL can negatively impact quality of life, with patients experiencing decreased body confidence, reduced physical activity, altered limb sensation, fatigue, and psychological distress [3]. There is marked interest in the prevention of these sequelae and in reduction in harm from breast cancer treatments more broadly, as more women than ever survive their breast cancer diagnosis [4]. Some proposed interventions for preventing BCRL include early postoperative physiotherapy, manual lymphatic drainage, compression sleeves, and other surgical interventions; however, there is no clear consensus on their effectiveness alone or in combination [2,3]. Studies have shown that many women who undergo breast cancer surgery experience uncertainty on how to prevent BCRL; adherence to some interventions, such as assigned exercises, can prove difficult [3].

The use of compression sleeves following breast cancer surgery has been proposed as a prophylactic treatment to prevent BCRL. Compression sleeves may provide a less invasive and manageable intervention to prevent BCRL. The body of knowledge on lymphedema management is extensive; however, there is a paucity of studies addressing emerging preventive measures. While there is significant interest in non-invasive approaches and patient-reported outcomes of such interventions, there has not yet been a systematic review of this intervention for BCRL. The current literature consists of a variety of observational and control studies that explore the effectiveness of compression garments in reducing risk of BCRL. Some preliminary studies have shown that compression garments worn in the subclinical phase of lymphedema can decrease risk of development of BCRL [2]. In addition, there is conflicting evidence on the type of compression sleeve that should be used in prevention and the pressure level of the garment [5]. While there have been extensive systematic reviews on multimodal preventative strategies for BCRL and on interventions to treat BCRL, there are no current systematic reviews that specifically address the effectiveness of compression garments in preventing BCRL.

## 2. Methods

### 2.1. Research Question and Objectives

This review aims to address the following question: in patients receiving axillary surgery for treatment of breast cancer (P), does the use of a postoperative prophylactic compression sleeve (I), compared to standard care, no sleeve, or placebo (C), reduce risk of developing ipsilateral breast cancer-related lymphedema (BRCL) (O)? The primary objective is to evaluate the effectiveness of prophylactic arm compression sleeves in reducing the incidence rate of arm lymphedema in patients who have received breast cancer surgery. The secondary objective is to evaluate how change in arm volume and quality of life are impacted by the use of prophylactic compression sleeves for BCRL. 

### 2.2. Search Strategy

This systematic review was conducted in accordance with the Preferred Reporting Items for Systematic Reviews and Meta-Analyses (PRISMA) guidelines. A publicly accessible review protocol was registered with the Open Science Framework (OSF; Registration DOI: 10.17605/OSF.IO/7K26F). A comprehensive search of electronic databases, including MEDLINE and Embase, was performed using the detailed search strategies outlined in Table A1 and Table A2, with combined terms related to “breast cancer,” “surgery,” “lymphedema,” and “compression sleeves.” The protocol includes full search strings, predefined inclusion and exclusion criteria, and a data-handling plan to ensure methodological transparency and reproducibility. The detailed search strings are provided in Appendix A (Table A1 and Table A2).

### 2.3. Inclusion and Exclusion Criteria

#### 2.3.1. Types of Participants

Trial participants of studies included in the review must have had either invasive or in situ carcinoma of the breast and undergone axillary surgery. Studies were excluded if patients had primary lymphedema or known pre-existing lymphedema at the onset of the trial. Studies with participants having had prior surgery or radiation therapy for any other indication to the head, neck, upper limb, or trunk were also excluded. 

#### 2.3.2. Types of Studies

This review included only randomized controlled trials (RCTs) that evaluated the effectiveness of prophylactic compression sleeves in preventing breast cancer–related lymphedema following surgery involving axillary intervention. Non-randomized studies, observational designs (e.g., cohort or case–control studies), case series, case reports, and conference abstracts were excluded. No restrictions were placed on publication year or country of origin. There was no minimum or maximum limit on the number of participants per study.

#### 2.3.3. Types of Intervention

Trials were included if the intervention was the use of compression sleeves/garments worn on the ipsilateral arm following breast cancer surgery. The intervention must have been administered no later than a month following breast cancer surgery. Sleeves had to be worn on a minimum daily basis for a period of at least 6 months. Studies must have had a control arm that was either described as a placebo, standard of care, or no intervention.

#### 2.3.4. Types of Outcome Measures

Trials were only included if the incidence rate of lymphedema development within 2 years was reported. Trials must have also reported on change in limb volume, measured at baseline and at follow-up. Studies were included if they described a qualitative assessment of quality of life, measured both at baseline and follow-up with a QOL tool. 

In consultation with an expert and on review of the literature, the definition of lymphedema was qualified as a >10% increase in arm volume measurement. Minimal important difference in health-related quality of life tools was interpreted referencing previously published studies in breast cancer patients [6]. 

Refer to Table 1 for the full inclusion and exclusion criteria.

### 2.4. Selection of Studies

Search results were independently reviewed by two different authors (AM and AM). Publications from the database search were imported into Covidence, and duplicates were excluded. The initial screening involved reviewing the title and abstract of each publication and excluding based on eligibility criteria. A second independent screening was performed based on the full-text review of the publication. Any disagreements between authors on eligibility of publications were resolved through discussion until agreement was achieved.

### 2.5. Data Extraction and Management

Two authors (AM and AM) independently extracted data from each publication included in the review. Disagreement on data was dealt with by engaging in conversation until a consensus was reached. Review authors were not blinded to the authors or affiliations of the publications. The following data was extracted from each publication:

1. Source: Study ID, citation, contact details.
2. Eligibility: Confirmation of eligibility for review.
3. Methods: Study design, study duration, sequence generation, allocation sequence concealment, blinding.
4. Participants: Number of participants, age, diagnostic criteria, morbidity, date of study.
5. Interventions: Type of sleeve use, frequency of sleeve use, length of sleeve use, time of start of intervention, co-interventions.
6. Control: Type of control.
7. Outcomes: Follow-up time/frequency, type of outcome, unit of measurement, outcome definition.
8. Results: Number of participants in each arm, number of drop-outs/loss to follow-up, incidence of lymphedema within 2 years (for each arm), arm volume at baseline and follow-up, HRQOL ratings at baseline and follow-up.
9. Other: Significant conclusions/comments from authors.

### 2.6. Assessment of Risk of Bias in Included Studies

Two authors (AM and AM) independently reviewed all included studies for risk of bias using the Revised Cochrane Risk-of-Bias Tool (RoB2) for randomized trials [7]. This tool was used to assess the possible risk of bias due to the randomization/concealment process, deviations from the intended interventions, blinding, missing outcome data, measurement of the outcome, statistical analysis, and reported results [8]. The tool yielded an overall level of bias risk (low, high, or some concerns), as well as levels of risk for individual domains. Conclusions from the risk-of-bias analysis were described and summarized in a risk-of-bias table. When faced with disagreement, the two authors engaged in discussion until agreement was reached. A third review author or expert was consulted in situations where an agreement could not be between the two reviewing authors. 

### 2.7. Assessment of Certainty of Evidence

In order to assess the certainty of evidence included in this review, the Grading of Recommendations, Assessments, Development and Evaluation (GRADE) approach was used [9]. The GRADE approach considers the risk of bias, inconsistency, indirectness, imprecision, and publication bias to determine any possible uncertainties or limitations to the outcome evidence. The GRADE assessment was used to assess the incidence rate of lymphedema, change in limb volume, and health-related quality of life. Within each domain, the total GRADE score was downgraded one level if a serious concern was found within each domain or downgraded two levels if a very serious concern was found; the levels remained the same if no serious concern was found. After assessment of each domain, the final GRADE result was determined using one of the four following grades: High certainty, moderate certainty, low certainty, or very low certainty. 

### 2.8. Measures of the Effect of Methods

The primary outcome (incidence of lymphedema) was a dichotomous outcome.

If sufficient homogeneity was present, pooled odds ratios and 95% confidence intervals were to be calculated using the Mantel–Haenszel method under a random-effects model. However, due to missing data and heterogeneity in outcome reporting, a meta-analysis was not feasible, and a narrative synthesis was performed instead.

### 2.9. Synthesis and Dealing with Missing Data

Only studies with available data were included for analysis; data was assumed to be missing at random given the absence would be unlikely to be related to the outcome in question (ex. lymphedema or quality of life). Effort was made to contact the original investigators to request missing data. Statistical methods were used to estimate sample mean and standard deviations from the given sample size, median, and interquartile range to allow pooling of results among trials [10].

## 3. Results

**Figure 1:**
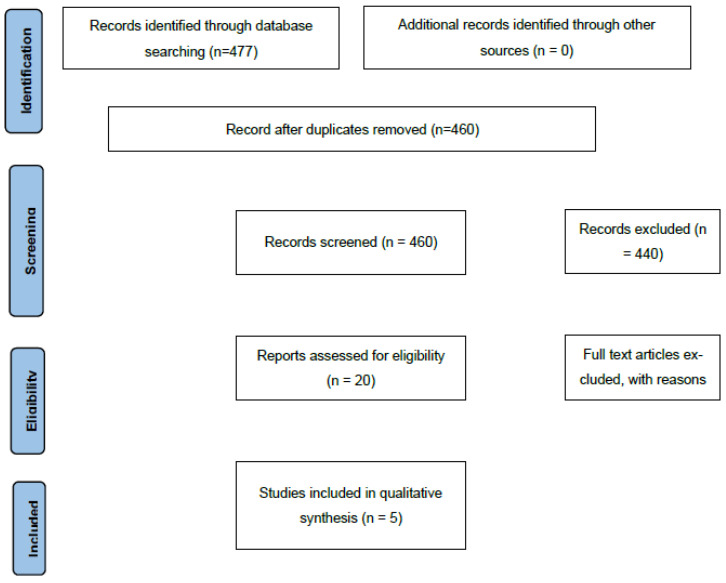
Flow diagram of identified and included publications.

Embase and Medline databases were searched using the Ovid interface, following a comprehensive search strategy developed with assistance from a Medical Librarian (G.B.). The full search strategies are presented in Table A1 and Table A2. Searches were limited to English-language publications and included all available records from database inception to 1 October 2025. To ensure thoroughness, grey literature sources such as ClinicalTrials.gov, the World Health Organization International Clinical Trials Registry Platform (WHO ICTRP), and conference abstracts indexed in Embase were also reviewed. Records were screened and sorted according to the PRISMA Flow Diagram (Figure 1).

A total of 477 records were identified, with 17 duplicates removed. Following title and abstract screening, 440 records were excluded. Twenty publications underwent full-text review, and several were excluded for not meeting eligibility criteria. One publication with missing data on all three outcomes was excluded after an unsuccessful attempt to contact the corresponding author. In total, five studies were included in the qualitative and quantitative synthesis (Table 2). When continuous outcomes were reported as medians with interquartile ranges (IQRs), these values were converted to means and standard deviations (SDs) using standard statistical imputation methods to maintain consistency across studies.

### 3.1. Risk of Bias in Included Studies

Overall, there was some concern of bias in all five studies included in this review and in all three outcomes examined in this review. Allocation was randomized in all five studies; however, there was no indication of concealment of allocation sequence or specific details on how randomization was performed, which presents some concern. There was an overall low risk of deviations from the intended intervention, even though most studies included unblinded participants and unblinded deliverers of the intervention. Three out of five publications showed low concern for missing outcome data. However, one study only had 83% of data available in the control arm, presenting a high concern of risk of bias. There was low to some concern for risk of bias due to measurement of the outcome.

All studies included an appropriate method of measuring the outcome and no difference in measurement strategies between intervention groups. However, most of the studies did not indicate whether outcome assessors were blinded or not. There was also low to some concern for risk of bias due to selective reporting; since most data analysis plans were specified a priori, there were singular outcome measures within each outcome domain and a singular statistical analysis plan. See Table 3 for a summary of risk of bias for each publication and each outcome.

### 3.2. Primary Outcome: Development of Lymphedema

All studies reported data on the development of lymphedema at defined postoperative time points. The five randomized controlled trials included a total of 1004 patients. Lymphedema measurements were taken at 18 months post-surgery in Paskett (2020) [12], at 1 year in Ochalek (2017) [2], at 2 years in Ochalek (2018) [11], at 24 months in Bundred (2023) [14], and at 12 months in Paramanandam (2022) [13].

A GRADE risk-of-bias assessment indicated significant concerns in the domains of randomization and missing outcome data, particularly in [11], which had a high drop-out rate and lacked a description of how this was handled or whether sensitivity analyses were conducted. Lesser concerns were noted for deviations from intended interventions, outcome measurement, and reporting bias. Overall, the certainty of evidence was rated low.

Across studies, the evidence suggests that prophylactic use of compression sleeves in the postoperative period does not result in a substantial reduction in the risk of developing lymphedema. Smaller studies tended to report greater benefits, highlighting the possibility of early-study bias, but the larger trials showed minimal measurable effect.

### 3.3. Secondary Outcome: Edema Volume (Lymphedema Severity)

All five studies reported on ipsilateral arm volume changes at various postoperative time points; however, incomplete data limited direct comparisons across trials. Two studies [2,11] reported both absolute arm volume (mL) and excess volume (“edema volume,” mL) compared to the contralateral arm. In contrast, Paskett et al. (2020) [12] defined lymphedema severity using changes in arm circumference, reporting only an estimated intergroup difference without specific units. Due to uncertainty, the study was not included in comparative analyses.

Further supporting evidence was provided by Bundred et al. (2023) [14], which defined lymphedema based on bioimpedance spectroscopy (BIS) and relative arm volume increase (RAVI). The incidence of arm swelling at 1 year was lower in the compression group compared to controls [13]:

- BIS-based swelling: 42% vs. 52% (HR = 0.61; 95% CI 0.43–0.85; *p* = 0.004).
- RAVI-based swelling: 14% vs. 25% (HR = 0.56; 95% CI 0.33–0.96; *p* = 0.034).

Similarly, Paramanandam et al. (2022) [13] measured lymphedema severity using limb volume changes at 3, 6, 9, 12, and 15 months, defining lymphedema as a >10% increase in limb volume. No significant between-group differences were observed at 12 months.

Although limited by small sample sizes and inconsistent measurements, collectively, the evidence suggests that prophylactic compression sleeves may reduce the severity and incidence of lymphedema in women undergoing breast cancer treatment. However, the certainty of evidence remains very low, underscoring the need for larger, standardized RCTs with longer follow-up durations.

### 3.4. Secondary Outcome: Health-Related Quality of Life (HRQOL) Section

All five included studies reported collecting HRQOL data via patient surveys, although only four provided analyzable results [2,11,13,14]. In [2,11], HRQOL data were reported at one year post-surgery. Compression therapy showed a slight numerical advantage in global health scores, but differences were not statistically significant and did not reach a clinically meaningful threshold.

Bundred et al. (2023) [14] reported changes in quality of life using the FACT-B and Trial Outcome Index (TOI) from pre-surgery to 12, 18, and 24 months. At 12 months, the median change in FACT-B +4 score was 0.5 for controls and 5 for the sleeve group (*p* = 0.36). For TOI, changes were 3.5 versus 4, respectively (*p* = 0.33). Functional Well-Being (FWB) scores improved significantly in the sleeve group (+6 vs. −1, *p* = 0.007), while Emotional Well-Being (EWB) changes were not significant (*p* = 0.24). These short-term improvements were observed at 12 months but were not sustained at 18 or 24 months.

Paramanandam (2022) [13] reported no significant group differences across four quality-of-life scales at baseline or during follow-up. Hazard ratios comparing compression versus control for time to meaningful change were non-significant across all scales, with comparable rates of change between groups [14].

Collectively, these findings suggest that prophylactic compression therapy does not provide sustained improvement in HRQOL among breast cancer patients post-surgery, although minor short-term benefits may occur within the first postoperative year.

### 3.5. Adherence to Intervention

Adherence to the intervention varied across studies. In the two earlier, smaller trials, compliance with sleeve use was high—96% in Ochalek (2017) [2] and even higher in Ochalek (2018) [11]. In contrast, the largest study by Paskett (2020) [12] reported that only 31% of participants wore the compression garments as prescribed. Bundred et al. (2023) [14] did not record exact adherence, while Paramanandam et al. (2022) [13] reported uncertainty as to the adherence protocol.

## 4. Discussion

This systematic review demonstrates that patients who use a postoperative prophylactic sleeve have similar lymphedema rates compared to the standard of care at two years following surgery. Five randomized controlled trials were included in this systematic review. Development of lymphedema is commonly defined as a change in limb volume greater than or equal to 10%; however, the means of volume measurement has varied among previous studies (including circumference, volumetry, perometry, and clinical assessment). During the first few years, a patient is felt to be at highest risk for lymphedema, and the use of a prophylactic sleeve is an attractive, low-cost, minimally invasive intervention when considering the morbidity and patient impact of lymphedema as an incurable (though manageable) disease. There have been no previous published systematic reviews or meta-analyses examining this intervention. 

For the primary outcome of risk of development of lymphedema, the analysis of the pooled data suggests that compression sleeves may result in little to no difference in decreasing the risk, with low certainty of the evidence. The secondary outcome of HRQOL score showed a very small effect on the risk of lymphedema. For the secondary outcome of edema volume, the intervention resulted in a reduction in edema volume, with a wide confidence interval likely explained by imprecision (using a smaller number of patients), and the certainty of evidence was deemed to be very low. Given the very low certainty of the evidence, compression sleeves may reduce edema volume, but the evidence is very uncertain.

The inclusion criteria across the five studies were consistently applied and enrolled patient cohorts that are representative of those with early breast cancer, which promotes generalizability of the results. However, in one study, patients in both study arms also received instruction from a trained lymphedema prevention educator to review guidelines for lymphedema care and prevention. As many clinical settings do not offer this expertise as part of standard care, this might limit the generalizability of the compression intervention in isolation.

In the context of this review, it is uncertain whether early prophylactic compression therapy confers any additional benefits to prevent BCRL. A clearer understanding of the precise physiologic role of external compression during healing of lymphatics would be beneficial before definitive conclusions may be drawn. Furthermore, although our review included studies measuring health-related quality of life, more specific tools do exist to measure this in the context of breast cancer and lymphedema in particular, which might improve the precision in assessment of this outcome. 

## 5. Conclusions

Overall, this study suggests that prophylactic compression sleeves result in little to no difference in the risk of lymphedema. However, the existing evidence base is limited in both sample size and methodological rigor, making it difficult to draw definitive conclusions. Further studies to better characterize the contribution, if any, of this specific intervention in the early postoperative period are warranted. High-quality randomized trials are needed, with attention to adequate randomization, use of validated and specific QOL instruments for treatment-related lymphedema, a priori subgroup analysis to address confounders such as BMI, extent of axillary surgery, and receipt of additional oncologic therapies. Standardized outcome measures across studies will also be essential to allow meaningful comparison and synthesis of findings. A multicenter study scope would ensure robust heterogeneity in local postoperative rehabilitation practices/programs. Understanding the reasons for documented poor adherence to the intervention in previous studies will be important for future trial design, overcoming limitations of use, and informing patient-centered treatment recommendations. Recommendations should also include the cost-effectiveness of the intervention, balancing this with morbidity and cost of lymphedema treatment.

## 6. Future Directions

The current evidence suggests that prophylactic compression sleeves offer limited benefit in preventing lymphedema and do not provide sustained improvements in health-related quality of life (HRQOL) among women undergoing axillary lymph node dissection for breast cancer. Several areas warrant further investigation:

1. **Targeted Populations and Risk Stratification:** Future studies should explore whether certain subgroups, such as patients with higher body mass index (BMI), extensive nodal involvement, or predisposing comorbidities, may derive greater benefit from prophylactic compression. Personalized risk-based approaches may optimize the use of compression garments.
2. **Longer-Term Follow-Up:** Existing trials have follow-up periods ranging from 12 to 24 months. Extended follow-up is needed to determine whether delayed-onset lymphedema or late changes in HRQOL occur beyond two years post-surgery.
3. **Standardization of Lymphedema Assessment:** Variation in outcome measures, including arm volume, RAVI, and bioimpedance spectroscopy (BIS), limits comparability across trials. Future studies should adopt standardized, validated measures and consistent thresholds for clinical and subclinical lymphedema.
4. **Adherence and Intervention Optimization:** Adherence to compression sleeve use varied widely, with some large trials reporting low compliance. Strategies to improve adherence—through patient education, comfort-focused designs, or digital monitoring—should be incorporated and evaluated, as real-world effectiveness may differ from trial efficacy.
5. **Quality-of-Life Outcomes:** Short-term improvements in Functional Well-Being were observed in some studies but were not sustained. Future research should explore additional patient-centered outcomes, including psychosocial impact, upper-limb function, and work-related limitations, to capture the broader impact of lymphedema prevention strategies.
6. **Alternative or Adjunctive Interventions:** Given the limited efficacy of prophylactic sleeves alone, research should investigate combined interventions, such as early physiotherapy, manual lymphatic drainage, or lifestyle modifications, to reduce risk and improve patient outcomes.
7. **Larger, High-Quality Trials:** The current body of evidence is limited by small sample sizes, variable adherence, and risk-of-bias concerns. Well-designed, adequately powered RCTs with rigorous randomization, robust adherence tracking, and pre-specified sensitivity analyses are needed to generate higher-certainty evidence.

Overall, future research should focus on optimizing patient selection, standardizing outcome measurement, and integrating behavioral and supportive interventions to meaningfully reduce the burden of lymphedema and improve HRQOL in breast cancer survivors.

## Figures and Tables

**Table 1:** List of exclusion and inclusion criteria.

**Inclusion Criteria**	English-language studies published after 2000. Participants with histologically confirmed invasive or in situ carcinoma of the breast. Participants who underwent axillary surgery (sentinel lymph node biopsy and/or axillary dissection). Baseline (preoperative) arm volume measurement obtained using a perometer, arm circumference, or both. Quantitative lymphedema measurement reported as the primary outcome. Minimum participant age >18 years. Intervention initiated within 1 month post-surgery. Follow-up period between 1 and 2 years after surgery. Longitudinal measurements collected during postoperative clinical follow-up. Studies including additional interventions alongside compression sleeves, provided co-interventions are consistent across study arms. Studies with blinded or unblinded investigators and participants
**Exclusion Criteria**	Participants with primary or pre-existing lymphedema. Participants with prior surgery or radiation to the head, neck, upper limb, or trunk for any other indication. Trials with >30% drop-out or loss to follow-up. Non-randomized trials. Trials in which the intervention was initiated after the onset of lymphedema or related symptoms.

**Table 2:** Description of RCT studies included.

Study	Design	Participants	Intervention	Comparison	Outcomes	Notes
Ochalek et al., 2017 [2]	RCT	N = 45; women with breast cancer preoperatively assigned to intervention.	12-month use of circular-knit Class I sleeves; worn 8–10 hours daily; educational leaflets provided on wear time and replacement.	No compression sleeves; physical activity only.	Follow-up at 12 months; limb volume (circumferential arm measurement); incidence of LE (>10% increase); HRQOL; physical activity.	Both groups received the same standardized physical activity program.
Ochalek et al., 2018 [11]	RCT	N = 45; women with breast cancer preoperatively assigned to intervention.	Circular-knit Class I sleeves; worn daily.	No compression sleeves; physical activity only.	Follow-up at 1 and 2 years; limb volume; incidence of LE (>10% increase); HRQOL.	Both groups received the same standardized physical activity program.
Paskett et al., 2020 [12]	RCT	N = 568; women > 18 years with breast cancer recruited from clinical sites.	Lymphedema Education and Prevention (LEAP): education, compression sleeve use, and physical activity.	Education only (no compression sleeves).	Follow-up at 6, 12, and 18 months; incidence of lymphedema (>10% increase); arm circumference; range of motion; adherence.	Quality of life outcomes not reported.
Paramanandam et al., 2022 [13]	RCT	N = 307; women > 18 years undergoing lymph node dissection.	Postoperative compression sleeve use; education on daytime wear.	Education only (no compression sleeves).	Follow-up at 3, 6, 9, 12, and 15 months; incidence of lymphedema (>10% increase); arm swelling.	No significant group differences for quality-of-life measures.
Bundred et al., 2023 [14]	RCT	N = 143; women with node-positive breast cancer postoperatively assigned to intervention.	12-month use of graduated compression garments; education on elevation, exercise, and self-massage.	No compression sleeves.	Follow-up at 1, 3, 6, 9, and 12 months; incidence of lymphedema (>10% increase); cellulitis incidence; BMI effect.	No significant differences in quality-of-life measures.

**Table 3:** Risk-of-bias assessment across included studies.

**Study**	**D1**	**D2**	**D3**	**D4**	**D5**	**Overall**
Ochalek 2017 [2]	Some concern	Low	Low	Some concern	Low	Some concern
Ochalek 2018 [11]	High	Some concern	High	Low	Some concern	Some concern
Paskett 2020 [12]	Some concern	Low	Low	Some concern	Low	Some concern
Paramanandam 2022 [13]	Some concern	Some concern	Some concern	Low	Low	Some concern
Bundred 2023 [14]	Low	Some concern	High	Some concern	Low	Some concern
Legend:						
**Low**		**Some concern**			**High**	

## Data Availability

All data extracted from included studies are available upon reasonable request.

## Appendix A

**Table A1:** List of search terms used for Ovid MEDLINE(R).

	Database: Ovid MEDLINE(R) Date of Search: 1 October 2025
1	exp Breast Neoplasms/
2	exp Mastectomy/
3	(breast adj1 cancer adj1 (surge * or treat *)).mp.
4	(mammary and carcinom *).mp.
5	exp Breast Cancer Lymphedema/
6	exp Surgical Oncology/
7	exp Carcinoma, Lobular/or Carcinoma/or exp Carcinoma, Ductal, Breast/or exp Breast Carcinoma In Situ/
8	exp Postoperative Care/
9	or/1-8
10	(compres * adj1 (sleeve * or garment * or band * or treat * or therap * or wrap * or stock *)).mp.
11	(arm and compres *).mp.
12	or/10-11
13	9 and 12
14	randomized controlled trial.pt.
15	controlled clinical trial.pt.
16	randomized.ab.
17	placebo.ab.
18	clinical trials as topic.sh.
19	randomly.ab.
20	trial.ti.
21	14 or 15 or 16 or 17 or 18 or 19 or 20
22	exp animals/not humans.sh.
23	21 not 22
24	13 and 23

**Table A2:** List of search terms used for Embase.

	Database: Embase Date of Search: 1 October 2025
1	breast cancer/co, dm, rt, si, su, th [Complication, Disease Management, Radiotherapy, Side Effect, Surgery, Therapy]
2	cancer patient/
3	compression therapy/
4	compression.ab.
5	lymphedema/co, pc, si [Complication, Prevention, Side Effect]
6	breast cancer-related lymphedema/co, dm, pc, si, th [Complication, Disease Management, Prevention, Side Effect, Therapy]
7	arm edema/co, pc, si [Complication, Prevention, Side Effect]
8	arm swelling/co, pc, si [Complication, Prevention, Side Effect]
9	1 or 2
10	3 or 4
11	5 or 6 or 7 or 8
12	9 and 10 and 11
13	limit 12 to (english language and yr = “2004 -Current”)
